# Research on the application of a multi-model cascaded deep learning framework in the pathological diagnosis of osteosarcoma

**DOI:** 10.3389/or.2025.1592408

**Published:** 2025-11-12

**Authors:** Hui Yao, Mengxue Yang, Xin Jiang, Hao Jia, Tao Sun, Molin Li, Taiping Wang, Xuefeng Tang

**Affiliations:** 1 Department of Pathology, Chongqing General Hospital, Chongqing University, Chongqing, China; 2 Chongqing Medical University, Chongqing, China; 3 Department of Pathology, Zhaotong First People’s Hospital, Zhaotong, Yunnan, China; 4 Hangzhou Medipath Intelligent Technology Co., Ltd., Hangzhou, Zhejiang, China

**Keywords:** osteosarcoma, multi-model cascaded deep learning framework, pathological diagnosis, vision mamba model, adolescent

## Abstract

**Introduction:**

Osteosarcoma is the most common malignant tumor of bone tissue in adolescents, and precise pathological diagnosis is the primary foundation for establishing the most effective treatment plan. The pathological evaluation of tumor necrosis after chemotherapy is crucial for assessing therapeutic efficacy in osteosarcoma patients. However, pathologists often face several challenges during the diagnosis and evaluation process.

**Methods:**

To address these needs, we designed and developed a multi-model cascaded deep learning framework utilizing an advanced Vision Mamba (ViM) model as the core network architecture. The study employed one of the most comprehensive osteosarcoma datasets, sourced from: (1) real-world data from 68 osteosarcoma patients collected at Chongqing General Hospital, and (2) publicly available osteosarcoma assessment data from the University of Texas Southwestern/UT Dallas. Pathological images were annotated using the Palgo pathology image artificial intelligence self-training platform according to algorithm requirements. A triple verification mechanism of annotation, review, and archiving was implemented, and Palgo’s integrated interactive algorithm correction mechanism was used to continuously refine the data annotation process.

**Results and Discussion:**

The model demonstrated Dice coefficient values of 0.83 or higher in tumor segmentation, osteosarcoma osteoid matrix segmentation, necrotic area segmentation, lung metastatic tumor segmentation, and lung metastatic osteoid matrix segmentation. For necrosis classification, overall osteosarcoma subtypes, and localized osteosarcoma subtypes, the area under the receiver operating characteristics curve (AUC), sensitivity, specificity, positive predictive value (PPV), and negative predictive value (NPV) all exceeded 90%. The proposed model exhibited excellent performance, indicating high potential for future clinical application in osteosarcoma patients. This framework shows promise for enhancing the precision and efficiency of pathological diagnosis and evaluation in osteosarcoma management.

## 1 Introduction

Osteosarcoma is a rare cancer, but it is the most common malignant bone cancer primarily affecting individuals between the ages of 10 and 30, making it the third most common cancer among children and adolescents (1, 2). Annually, there are approximately 4.4 cases per million children worldwide (3). The disease can be classified in various ways, including primary and secondary types. Primary osteosarcoma accounts for 75% of cases (3), typically occurring in children and young adults and presenting as abnormal bone growth, while secondary osteosarcoma is more common in adults with mature bones, usually triggered by another disease. Primary osteosarcoma also varies in form, with common types including intramedullary, parosteal, and periosteal osteosarcomas (4). In addition, osteosarcomas can be categorized as conventional central osteosarcoma (commonly featuring osteoblastic, chondroblastic, and fibroblastic cells), vascular spread type, intrabony type, and small cell osteosarcoma (5).

The most common sites for osteosarcoma are the femur (42%), tibia (19%), and humerus (10%) (6). Those located at the distal femur and proximal tibia have a survival rate of 50%–65%, however 25%–50% of patients with initial metastases succumb to pulmonary metastasis (7). Although the progression of localized and distant osteosarcoma metastases is slow, the presence or absence of metastasis is an important prognostic factor (8). Additionally, cancer cells often exhibit abnormal apoptotic mechanisms that promote tumor development and pose challenges for the effective treatment of tumors due to the resulting resistance to treatment (9). Thus, early detection and accurate prediction of treatment responses in osteosarcoma are crucial for improving patient prognosis.

Moreover, traditional pathological diagnosis relies heavily on the experience and expertise of pathologists, which is not only time-consuming but also susceptible to subjective judgment. With the explosive growth of medical image data, the limitations of manual analysis are becoming increasingly apparent. Computer-assisted detection (CAD) is essential to aid clinicians in examining histopathological images. CAD-based analysis of histopathological images is also a challenging field within biomedical image analysis (10). Recent studies based on medical data have shown that deep learning (DL) can be used to extract and analyze medical image information with great success (11, 12).

The use of artificial intelligence (AI) has revolutionized osteosarcoma research, with both traditional machine learning and DL techniques achieving significant advancements. For instance, traditional methods, such as random forest (RF) and support vector machines (SVMs) have demonstrated promising accuracy in classifying osteosarcoma based on metabolomic and histopathological data (26, 28). In addition, DL models, including convolutional neural networks (CNNs) and generative adversarial networks (GANs), have shown exceptional performance in tumor classification and segmentation, achieving detection accuracies as high as 96% (32, 40). Additionally, frameworks such as UNet [53] and Deeplab [57] have further enhanced segmentation precision, even with limited datasets.

`Recent advances in transformer-based models, such as the Vision Transformer (ViT), have introduced self-attention mechanisms that excel in capturing global image features (21). The Vision Mamba (ViM) model, a state-of-the-art alternative, reduces computational demands while maintaining high performance, operating at 2.8 times the speed of traditional ViT models and consuming 86.8% less GPU memory (23). Prognostic studies incorporating AI have also identified critical biomarkers and predictive models, such as DeepSurv, which outperform classical methods like Cox regression analysis in survival prediction (45). These advancements underscore the potential of AI in improving both diagnostic accuracy and prognostic evaluation in osteosarcoma studies.

In this study, we implemented the cutting-edge DL model Mamba (13), using it across various domains, including classification and image segmentation. Within the realm of DL, frameworks such as CNNs (14–20) and ViT (21) have achieved remarkable outcomes. CNNs excel at processing local patterns and textural details, but struggle with capturing the broader context and long-range dependencies within images. On the other hand, the transformer architecture (22), particularly the ViT, has outperformed in numerous visual tasks, leveraging its prowess in handling long-distance dependencies and sequential data processing. However, ViT models usually require extensive datasets for training and are computationally expensive. In this study, we used the advanced ViM model as our core network architecture, which operates at 2.8 times the speed of contemporary ViT models while consuming approximately 86.8% less GPU memory, thus offering an efficient alternative that mitigates the heavy computational demands of traditional transformer models (23).

Additionally, since digital pathological images are obtained by scanning histopathological images, whole slide imaging includes a vast amount of data, with a single histopathological unit containing numerous cells (24). Handling such big data with a single model presents significant challenges. Our study uses a multi-model cascading approach to effectively tackle this issue. In the low-magnification view, the first-stage model completes a general feature analysis and preliminary localization of tumor regions. Then, in the high-magnification view, subsequent models perform a detailed observation of specific tumor cell subtypes, enhancing diagnostic precision. In addition, through a branching structure, it is possible to parallel process additional pathological features, such as the diagnosis of lung metastasis, thus achieving a more comprehensive disease analysis and evaluation. This multi-model cascading and branching approach not only fully leverages the strengths of different models to enhance the efficiency and accuracy of processing large-scale pathological images, but also allows for flexible adjustments according to specific tasks, offering new possibilities for the intelligent diagnosis of complex diseases like osteosarcoma.

The structure of this article is as follows: After this introduction section, Section 2 describes the data collection and labeling process used in this study, and details how we use a multi-model cascading DL framework to detect osteosarcoma; Section 3 describes the analysis of the experimental results of osteosarcoma detection; finally, Section 4 discusses existing issues and directions for improvement.

## 2 Materials and methods

### 2.1 Materials

In this study, we used one of the most comprehensive datasets of osteosarcoma patients, obtained from two key sources: 1. real-world data from 68 osteosarcoma patients collected at the Chongqing General Hospital (from May 2012 to March 2022), and 2. publicly available osteosarcoma assessment data from the University of Texas (UT) Southwestern/UT Dallas, which includes records of 50 patients treated at Children’s Medical Center Dallas between 1995 and 2015 (https://wiki.cancerimagingarchive.net/pages/viewpage.action?pageId=52756935, accessed January 10, 2023) (25).

The clinical characteristics of 68 osteosarcoma patients are shown in Table 1, among whom 32 patients received preoperative chemotherapy. The chemotherapy regimens are summarized in Table 2. The inclusion and exclusion criteria for tissue slides were as follows:

**TABLE 1:** Clinical characteristics in osteosarcoma patients (n = 68).

Characteristic	Cases	%
Sex		
Male	41	60.3
Female	27	39.7
Age (years)		
≤18	30	44.1
>18	38	55.9
Tumor size		
≥5 cm	39	57.4
<5 cm	29	42.6
Clinical stages (Enneking stages)		
IA	1	1.5
IB	2	2.9
IIA	25	36.8
IIB	26	38,2
III	14	20.6
Region		
Thighbone	35	51.5
Tibia	13	19.1
Pelvis	8	11.8
Shoulder/Humerus	4	5.9
Jaw	3	4.4
Fibula	2	2.9
Vertebra	1	1.5
Extraosseous	2	2.9

**TABLE 2:** Chemotherapy regimens for osteosarcoma patients (n = 32).

Chemotherapy regimens	Drug	Cases (%)
MAP	Methotrexate; Doxorubicin; Cisplatin	20 (62.5)
AP	Doxorubicin; Cisplatin	6 (18.7)
AD	Pirarubicin; Nedaplatin	2 (6.3)
GP	Gemcitabine; cisplatin	2 (6.3)
IP	Ifosfamide; cisplatin	1 (3.1)
IE	Ifosfamide; etoposide	1 (3.1)

#### 2.1.1 Inclusion criteria

1. Cases of osteosarcoma confirmed by histology and treatment efficacy.
2. Complete clinical data available.
3. Adequate tissue samples obtained.
4. Decalcified tissue, with well-preserved fixation, and satisfactory staining results.

#### 2.1.2 Exclusion criteria

1. Specimens containing disputed elements where a diagnostic consensus could not be reached.
2. Poor-quality slides due to issues during the preparation process, such as overly thin or thick tissue sections, knife marks, cell distortion, or unsatisfactory staining.

To ensure the adequacy of the training data and prevent overfitting, we used not only conventional data augmentation techniques, such as color perturbation, rotation, and scaling, but also a pathology-specific large-tile random cropping method, provided by the Palgo platform, during training.

Palgo Pathology Image Artificial Intelligence Self-Training Platform (https://www.palgo.com.cn/) is an advanced AI tool for automated pathology image analysis. It integrates deep learning algorithms, annotation tools, and automated model optimization processes, enabling efficient training and deployment of customized AI models for pathology tasks. Pathology images were uploaded to the platform in standardized formats (e.g., JPEG or TIFF) and preprocessed using built-in augmentation tools. Model training was tailored to the specific task, employing a transfer learning approach with a pre-trained model, further fine-tuned on a labeled dataset to enhance task-specific performance. Automated hyperparameter tuning optimized learning rates and batch sizes, reducing manual intervention. Evaluation used a 20% hold-out test set with metrics including Dice Similarity Coefficient and Intersection-over-Union (IoU). The platform’s self-training capabilities enabled customization of convolutional neural network (CNN) architectures for the specific task. Its interactive annotation tool expedited high-quality training data creation, while the visual analytics dashboard allowed real-time monitoring of performance. The Palgo platform was pivotal in achieving efficient and accurate renal pathology image segmentation, reducing manual annotation efforts while maintaining high performance. These methods were crucial in ensuring the robustness and adequacy of the training data, as detailed below.

From the Chongqing General Hospital, we collected data from 68 osteosarcoma patients, including 40 cases of osteoblastic osteosarcoma, 13 cases of chondroblastic osteosarcoma, 7 cases of fibroblastic osteosarcoma, and 8 cases of other rare osteosarcoma subtypes. In total, we obtained 128 whole slide images (WSIs) stained with hematoxylin and eosin (H&E) staining, including 9 lung metastasis samples. The data statistics used in each cascaded algorithm are provided in Table 3. For example, in the tumor region segmentation algorithm, the training set included annotations for 2,035 tumors, while the test set had annotations for 150 tumors. Similarly, in the tumor cell segmentation algorithm, four types of cells were labeled: osteoblastic, chondroblastic, fibroblastic, and other cells. Osteoblastic cells were the most prevalent, with 23,799 labeled training targets and 327 test targets. For necrosis classification, the training set contained 1,248 non-necrotic samples and 244 necrotic samples, while the test set included 101 non-necrotic and 22 necrotic samples. More detailed statistics are given in Table 3.

**TABLE 3:** Details of data annotation for each algorithm.

Algorithm	Category	No. of annotated patches	
		Training	Testing
Tumor region segmentation	tumor cell	2035	150
Osteoid matrix segmentation	Osteoid matrix	3410	222
necrotic region segmentation	necrosis	157	13
pulmonary metastatic tumor segmentation	tumor cell	10	2
pulmonary metastatic osteoid matrix segmentation	Osteoid matrix	2908	107
Tumor cell segmentation	Osteoblastic osteosarcoma	23799	327
	chondroblastic osteosarcoma	2320	62
	Fibroblastic osteosarcoma	3718	360
	others	612	11
necrosis	necrosis	1248	101
	No necrosis	244	22
	non-tumor	420	86
OS overall subtypes	Osteoblastic osteosarcoma	348	14
	chondroblastic osteosarcoma	91	30
	Fibroblastic osteosarcoma	68	2
	others	175	74
OS local subtypes	Osteoblastic osteosarcoma	231	31
	chondroblastic osteosarcoma	179	23
	Fibroblastic osteosarcoma	102	16
	others	34	2

All annotations were performed by three mid-level pathologists from Chongqing General Hospital, each with over 5 years of diagnostic experience. These annotations were then reviewed by two senior pathologists with over 10 years of experience to ensure accuracy. It is worth noting that each WSI has a resolution of approximately 100,000 × 100,000 pixels, but for training purposes, we used smaller image tiles, each with size of 1,024 × 1,024 pixels. To further increase the dataset, we used the random scaling and cropping method of Palgo, which generates around 100 random tiles from the effective regions of each WSI during each round of training. Compared to traditional fixed-tile methods, this approach ensures that the characteristics of each tile differ throughout the training process, while also allowing for multiple labeled regions to be processed in a single WSI. For example, in the tumor region segmentation task, using 128 WSIs resulted in approximately 12,800 tiles per training round. The labeling process also used the tiling approach, with annotations distributed randomly across the tiles.

Additionally, we used the publicly available osteosarcoma dataset from the UT Southwestern/UT Dallas to evaluate viable and necrotic tumors. This dataset consists of H&E-stained osteosarcoma histopathological images and includes records of 50 patients treated at Children’s Medical Center Dallas between 1995 and 2015. It is one of the most commonly used datasets in the research community. The dataset comprises 1,144 images, each sized 1,024 × 1,024 pixels, and was annotated by two clinical experts. In our study, we directly used the fixed-tile method from this dataset.

### 2.2 Algorithms

The aim of this study was to develop and validate a multi-model cascaded DL framework for the intelligent diagnosis of osteosarcoma from pathological images. By combining state-of-the-art DL models, particularly the ViM network and ViM UNet (ViM-UNet) architecture, we have constructed a comprehensive analysis system capable of accurately identifying and segmenting key features in osteosarcoma pathology images, including viable tissue regions, tumor areas, bone-like matrix, necrotic zones, as well as performing fine classification of tumor cells and detecting lung metastasis tumors.

#### 2.2.1 Classification models

**FIGURE 1:**
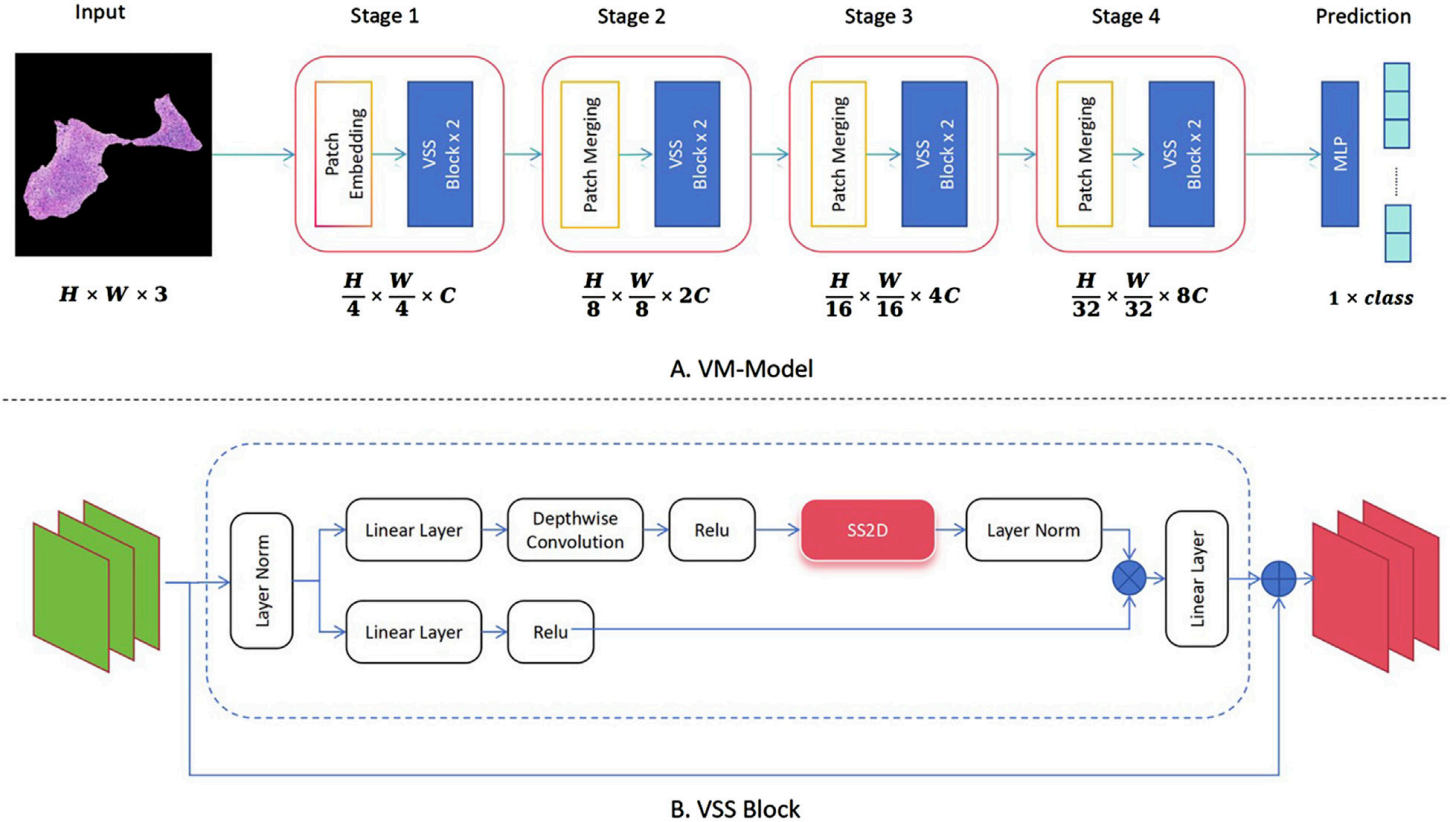
Implementation of the classification algorithm using Vision Mamba (ViM) as the feature extraction network. **(A)** Complete classification network structure of the ViM model. **(B)** Specific composition of the core VSS block module.

The analysis system developed in this study incorporates the latest ViM network as a feature extractor for image classification. As shown in Figure 1A, different from the original ViM model, in this study the network structure is divided into four stages, using patch merging between each stage to reduce the feature map size and double the channel numbers from the original. Notably, between the original input image and Stage 1, this study uses the same patch embedding structure as that in a Swin transformer network, which directly reduces the feature map to 1/4 of its original size (26, 27). The Virtual Storage Software (VSS) block is the core module in each stage, as depicted in Figure 1B, with the most important two-dimensional (2D) Selective Scan (SS2D) adopting a structure similar to that of ViM-UNet (28). In our study, this structure effectively extracts classification features and achieves good classification results. In addition, it serves as a consistent backbone and encoder for the subsequent segmentation model.

#### 2.2.2 Segmentation models

**FIGURE 2:**
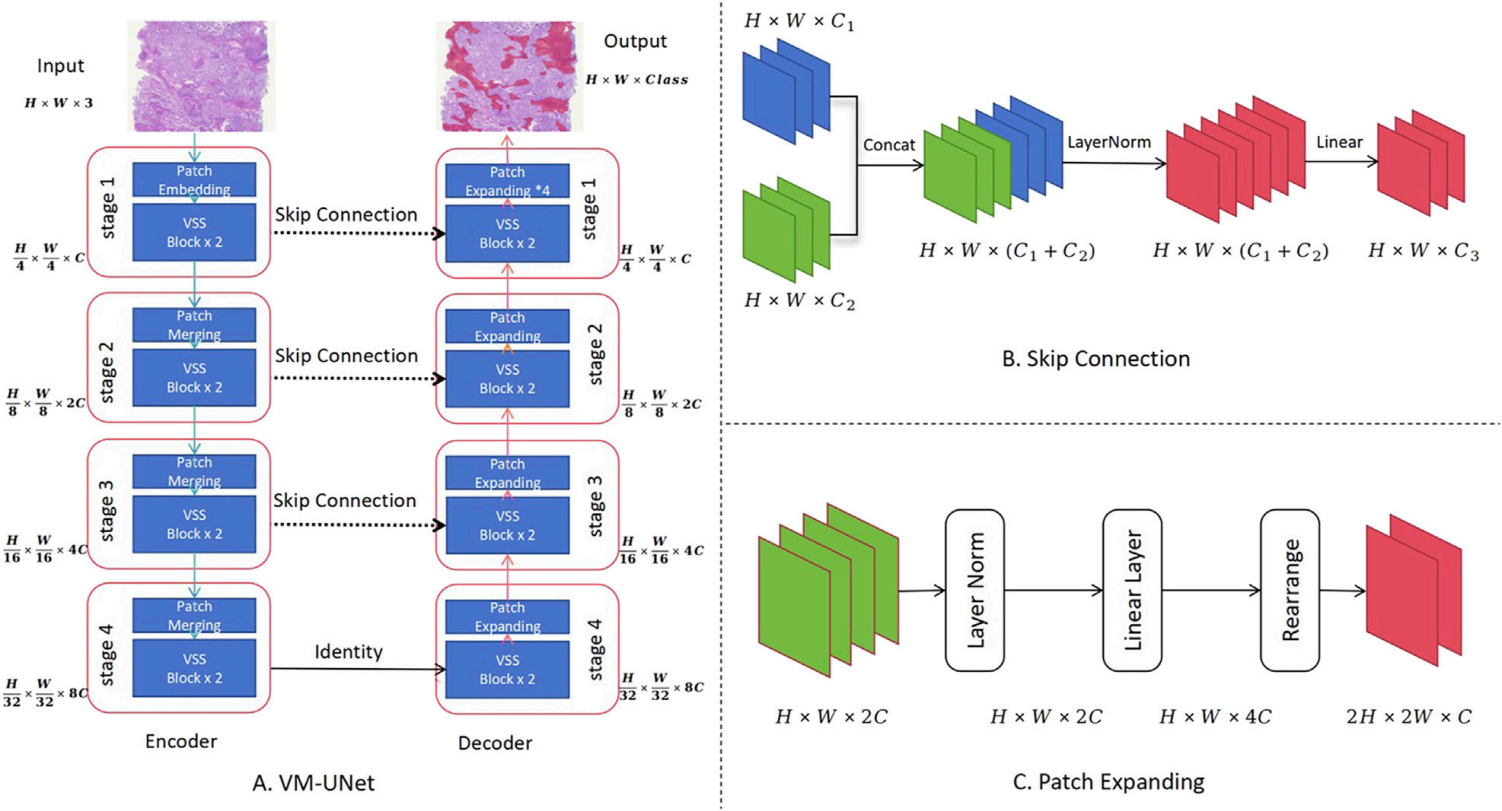
Use of the Vision Mamba UNet (ViM-UNet) as the image-based segmentation network. **(A)** The complete UNet structure is shown, including the encoder and decoder. **(B)** Diagram showing how the skip connection structure achieves feature fusion between the encoder and decoder. **(C)** Diagram showing how the patch expanding structure, actually implemented, achieves sampling and feature recovery.

In this study, the ViM network and ViM-UNet are combined to form the image-based segmentation network, as shown in Figure 2. The VSS block used in this study is the same as that used in the classification model shown in Figure 1A. Unlike ViM-UNet, in this study the commonly used skip connection structure is used in UNet to achieve feature fusion between the encoder and decoder, as shown in Figure 2B. Additionally, patch expanding is used as the upsampling module, with the structure detailed in Figure 2C. It must be noted that, in this study, in order to maintain consistency with the input feature map size, in the final stage of the decoder the patch expanding structure outputs four times the channels in the linear layer compared to before, thus transforming the feature map from (H × W×2C --> H × W × 16C). Another difference from ViM-UNet (28) is that due to the larger pixels of the pathology images compared to other medical images, a tile-based segmentation approach is used for prediction. This involves pre-scaling the images with fixed-scale proportional scaling, then dividing the scaled images into fixed-size tiles with overlap for full-image segmentation. Each tile is individually analyzed using the aforementioned network for prediction, and the results are then stitched back together. In addition, a linear distance-weighted fusion method is applied to the junctions for seamless integration.

#### 2.2.3 Model cascading

**FIGURE 3:**
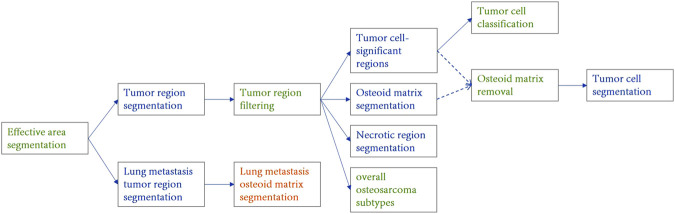
Structural diagram of the osteosarcoma cascaded model ensemble. The blue component represents the instance segmentation network, the yellow component represents the semantic segmentation network, and the green component represents the image classification or filtering module. Different stages operate at specific image resolutions (e.g., mpp = 5.0, 2.0, 0.23) and are connected via gated logic based on confidence and area thresholds. Detailed parameters are provided in Supplementary Table S2.

The Palgo platform was used in this study to design the model cascade strategy, as shown in Figure 3, achieving a gradual refinement of the extraction of pathological features from coarse to fine by performing the analysis at different resolutions and perspectives. The initial model swiftly identifies and locates key tissue regions at low magnifications, while subsequent models perform more detailed analysis and classification at higher magnifications to ensure comprehensive and accurate pathological diagnosis. The data flow within the cascade uses a “threshold + type gating structure” to automatically control the downstream algorithm data. For example, in the gate algorithm for excluding osteoid matrix, positions judged to be osteoid matrix within tumors are automatically removed before detailed analysis of tumor cells. Details of magnification levels (e.g., mpp = 5.0, 2.0, 0.23), input sizes, and gating thresholds used at each stage are comprehensively summarized in Supplementary Table S2.

In this study, tissue region localization and tumor area positioning are achieved at low magnifications, osteoid matrix segmentation and necrotic area localization at medium magnifications, and tumor cell identification and specific tumor type classification at high magnifications. The overall subtype classification for osteosarcoma is determined through a global view, while the localized osteosarcoma subtypes (in the tumor cell cascaded classification module) focus on the determination of the subtype of the most prominent tumor regions at high magnifications. Tumor cell segmentation further identifies and locates tumor cells based on their specific types.

To account for the histological heterogeneity of osteosarcoma, we designed two complementary subtype classification strategies in the cascade:

1. Overall subtype classification, which determines the dominant subtype by aggregating the total predicted area of each subtype across the entire slide;
2. Localized subtype classification, which determines the dominant subtype based on the number of positively predicted tiles for each subtype within high-magnification tumor cell regions.

The block-count-based localized subtype classification emphasizes discrete, high-grade foci (e.g., chondroblastic or fibroblastic areas) that, despite their small size, are clinically significant. Compared to area-based methods easily biased by large low-grade regions, this approach more accurately reflects the focal heterogeneity of aggressive subtypes and aligns with pathological assessment and clinical decision-making.

#### 2.2.4 Training methods

In this study, a multi-round weakly supervised training scheme with manual intervention was used, continuously optimizing and adjusting model performance by incorporating expert knowledge and feedback at different stages. Also, by combining data augmentation, transfer learning, and fine-grained annotation strategies, the limited pathological image resources are fully utilized to enhance the generalization ability and diagnostic accuracy of the model. Additionally, the initial model undergoes transfer learning after training on a large-scale dataset. The model uses the Adam optimizer and implements a learning rate warm-up strategy. The loss function used is the cross-entropy loss, applying the cross-entropy loss at each pixel for the segmentation task.

To improve methodological transparency and ensure reproducibility, the complete training configuration (including optimizer settings, learning rate schedules, and pathology-specific augmentation strategies) is described in Supplementary Text S1. In addition, task-specific hyperparameters such as input resolution, patch size, and batch size for each model are summarized in Supplementary Table S1.

### 2.3 Statistical analysis

In this study, a comprehensive statistical analysis was conducted to evaluate the performance of the proposed multi-model cascaded DL framework in the detection and classification of various pathological features of osteosarcoma. The evaluation metrics included the Dice coefficient, intersection over union (IoU), sensitivity, specificity, precision, recall, false positive rate (FPR), true positive rate (TPR), negative predictive value (NPV), positive predictive value (PPV), false discovery rate (FDR), false omission rate (FOR), and overall accuracy (ACC). These metrics provided a holistic assessment of the capability of the model in localizing and segmenting tumor regions, osteoid matrix, necrotic areas, tumor cells, and pulmonary metastatic regions, as well as in classifying necrosis and subtypes of osteosarcoma.

For segmentation tasks, the Dice coefficient values and IoU values were the primary metrics used to measure the spatial overlap between predicted regions and ground truth. Sensitivity, specificity, and precision were used to assess the detection performance for each segmented category, while the FPR and TPR quantified the trade-off between false positives and true positives. For classification tasks, metrics such as area under the receiver operating characteristics (ROC) curve (AUC), precision-recall (PR) curves, and confusion matrices were used to evaluate the accuracy of necrosis detection, overall classification of osteosarcoma subtypes, and classification of localized osteosarcoma subtypes.

The statistical analysis was performed on both training and test datasets, with multiple evaluations performed for each model to ensure robustness and consistency. Special attention was given to the challenges posed by the variability and complexity of datasets, such as the segmentation of small-scale tumor regions and highly overlapping cellular structures. To address these challenges, we analyzed discrepancies in Dice scores for smaller objects and investigated the impact of limited sample sizes on the classification accuracy. All statistical calculations were performed using Python libraries, including Scikit-learn, to ensure precise and reproducible results.

## 3 Results

We performed a statistical analysis of the effectiveness of detecting various features in osteosarcoma pathology images. The detection tasks included the localization and segmentation of tumor regions, osteoid matrix, necrotic areas, tumor cell regions within the tumor, and pulmonary metastatic tumor regions. Additionally, we also assessed the classification results for overall osteosarcoma subtypes, localized osteosarcoma subtypes, and the presence of necrosis. The performance of the models was comprehensively evaluated using metrics such as Dice coefficient, IoU, sensitivity, specificity, precision, recall, FPR, TPR, NPV, PPV, FDR, FOR, and ACC.

### 3.1 Localization and segmentation of tumor regions, osteoid matrix, necrotic areas, tumor cell regions within the tumor, and pulmonary metastatic tumor regions

**FIGURE 4:**
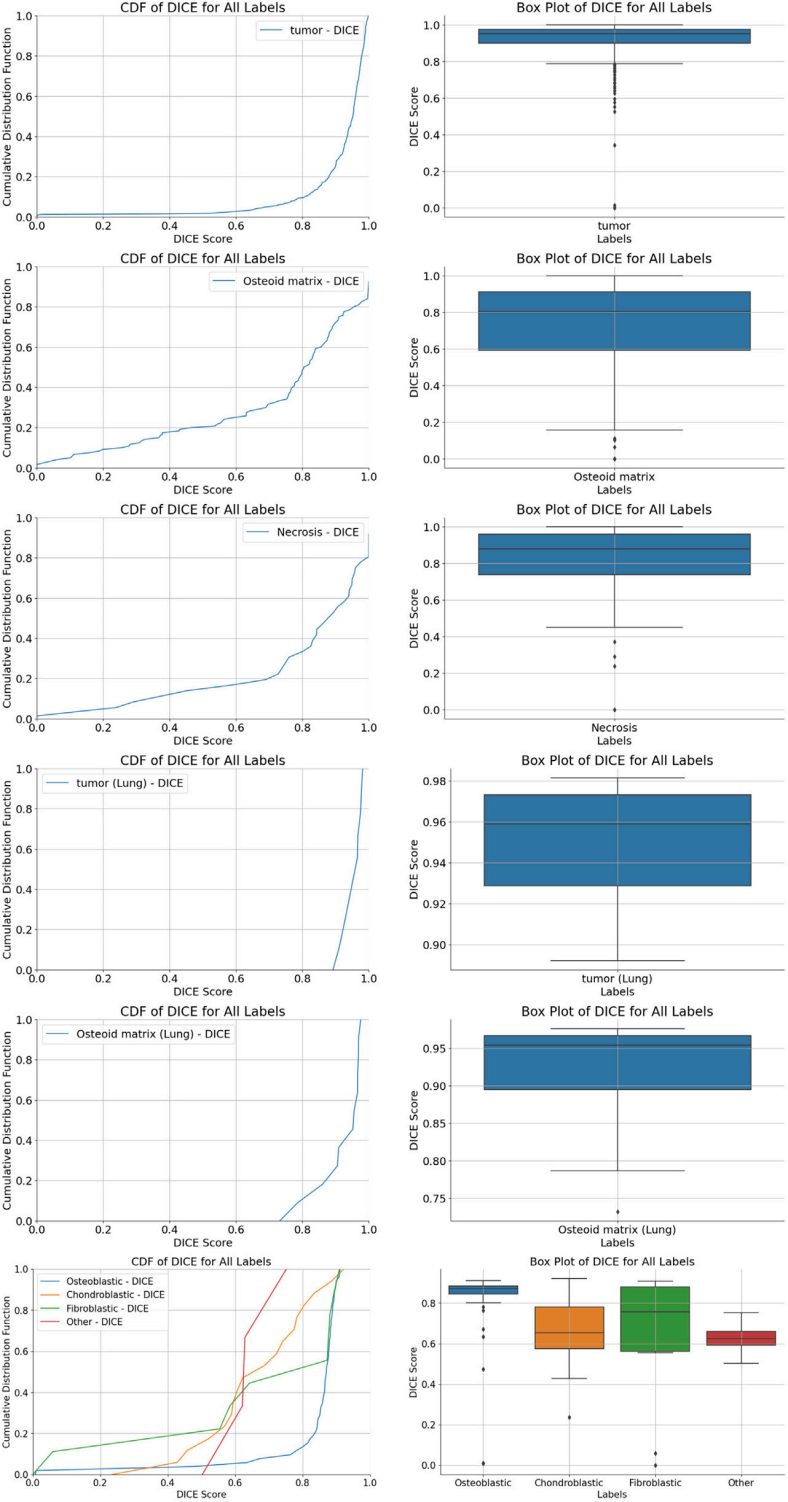
Cumulative distribution function (CDF) distribution of Dice coefficient values for various region segmentations. **(A,B)** tumor region segmentation; **(C,D)** osteoid matrix segmentation; **(E,F)** necrotic region segmentation; **(G,H)** pulmonary metastatic tumor segmentation; **(I,J)** pulmonary metastasis osteoid matrix segmentation; **(K,L)** tumor cell segmentation, along with boxplots of Dice coefficient scores for each algorithm.

We used five segmentation models to determine the localization and perform the segmentation of tumor regions, osteoid matrix, necrotic areas, tumor cells within the tumor, and pulmonary metastatic tumor regions (the performance of each model is shown in Figure 4). All models used the ViM-UNet architecture. The best results for tumor region segmentation were achieved with both primary osteosarcoma and pulmonary metastasis tumor segmentation attaining over 0.95 Dice correlation value (see Table 4; Figure 4 for details). However, the tumor segmentation results showed a relatively high FPR, suggesting that some small targets were detected, which, despite not significantly affecting overall detection, did increase the FPR. To address this issue, we incorporated a small tumor region filtering classification algorithm to filter out these small targets (referred to as the tumor region filtering model in Figure 3). Additionally, ViM-UNet showed excellent performance in necrotic region segmentation, achieving a Dice score of 0.942.

**TABLE 4:** Detailed values for tumor segmentation, osteosarcoma osseous matrix segmentation, necrotic area segmentation, lung metastasis tumor segmentation, lung metastasis osseous matrix segmentation, and tumor cell segmentation.

Label	DICE	IOU	Sensitivity	Specificity	Precision	Recall	FPR	TPR	NPV	PPV	FDR	FOR	ACC
tumor	0.9506	90.59%	94.00%	89.22%	96.15%	94.00%	10.78%	94.00%	83.84%	96.15%	3.85%	16.16%	92.76%
Osteoid matrix	0.8348	71.65%	76.74%	99.89%	91.53%	76.74%	0.11%	76.74%	99.63%	91.53%	8.47%	0.37%	99.52%
Necrosis	0.9420	89.04%	95.70%	97.44%	92.76%	95.70%	2.56%	95.70%	98.51%	92.76%	7.24%	1.49%	96.99%
tumor (Lung)	0.9525	90.93%	92.15%	99.23%	98.56%	92.15%	0.77%	92.15%	95.66%	98.56%	1.44%	4.34%	96.65%
Osteoid matrix (Lung)	0.9358	87.93%	95.46%	96.92%	91.77%	95.46%	3.08%	95.46%	98.34%	91.77%	8.23%	1.66%	96.53%
Osteoblastic	0.8332	71.42%	76.66%	99.20%	91.26%	76.66%	0.80%	76.66%	97.50%	91.26%	8.74%	2.50%	96.99%
Chondroblastic	0.7184	56.06%	60.51%	99.87%	88.39%	60.51%	0.13%	60.51%	99.38%	88.39%	11.61%	0.62%	99.26%
Fibroblastic	0.7270	57.11%	63.53%	99.75%	84.98%	63.53%	0.25%	63.53%	99.20%	84.98%	15.02%	0.80%	98.97%
Other	0.7166	55.83%	59.65%	99.98%	89.73%	59.65%	0.02%	59.65%	99.89%	89.73%	10.27%	0.11%	99.88%

**FIGURE 5:**
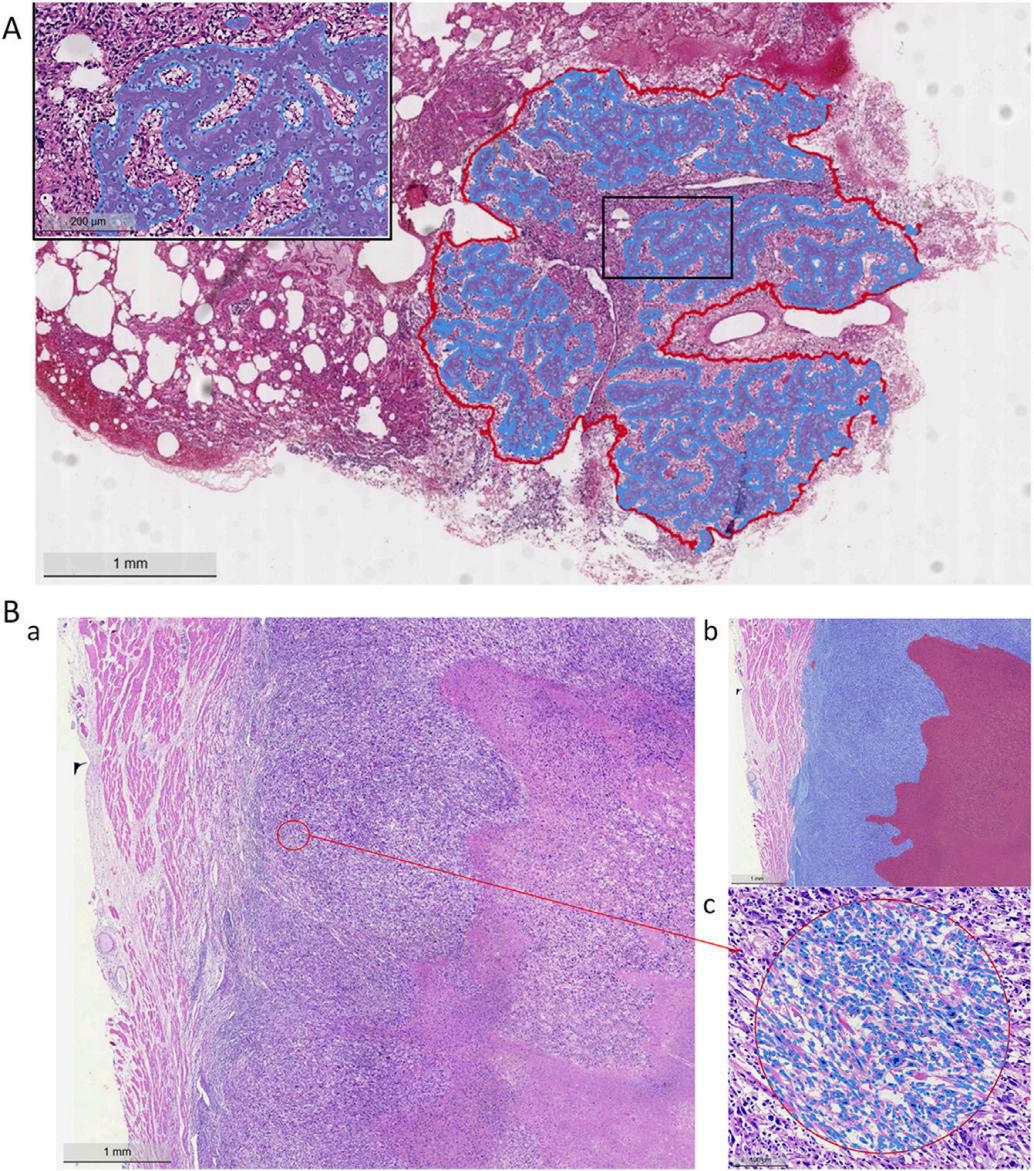
Model segmentation performance. **(A)** Lung metastasis tumor segmentation and osteoid matrix segmentation localization (red curve indicates the metastatic tumor area, blue curve indicates the osteoid matrix area). **(B)** Necrotic region segmentation and tumor cell identification (a: H&E staining image, b: blue area on the left indicates tumor cells, red area on the right indicates necrosis, c: tumor cell identification).

For osteoid matrix segmentation, the performance in pulmonary metastatic osteoid matrix (Dice score of 0.9358 was notably better than that in primary osteosarcoma (Dice score 0.8348). As shown in the upper part of Figure 5, the osteoid matrix features are highly distinguishable in pulmonary metastatic samples, making them easier to recognize. In primary osteosarcoma samples, where the pathology is more complex and there are many interfering structures like osteogenic tissue, periosteum, and cartilage, the segmentation task is more challenging. However, despite these complexities, the model still performed well in identifying osteoid matrix within osteosarcoma.

In the tumor cell segmentation task, the ViM-UNet model achieved accurate localization and subtype identification of osteoblastic, chondroblastic, and fibroblastic cells. As shown in Table 4 and Figure 4, the Dice scores for these subtypes were relatively lower (minimum 0.7184) compared to other segmentation tasks. This can be attributed to the small size of individual cells, which leads to higher sensitivity of the Dice metric to minor boundary deviations. Nevertheless, the model demonstrated high performance in terms of accuracy (minimum 97%), specificity, and precision. A detailed analysis of metric behavior for small target volumes is provided in Supplementary Text S2.

### 3.2 Classification results of overall osteosarcoma subtypes, localized osteosarcoma subtypes, and the presence of necrosis

**FIGURE 6:**
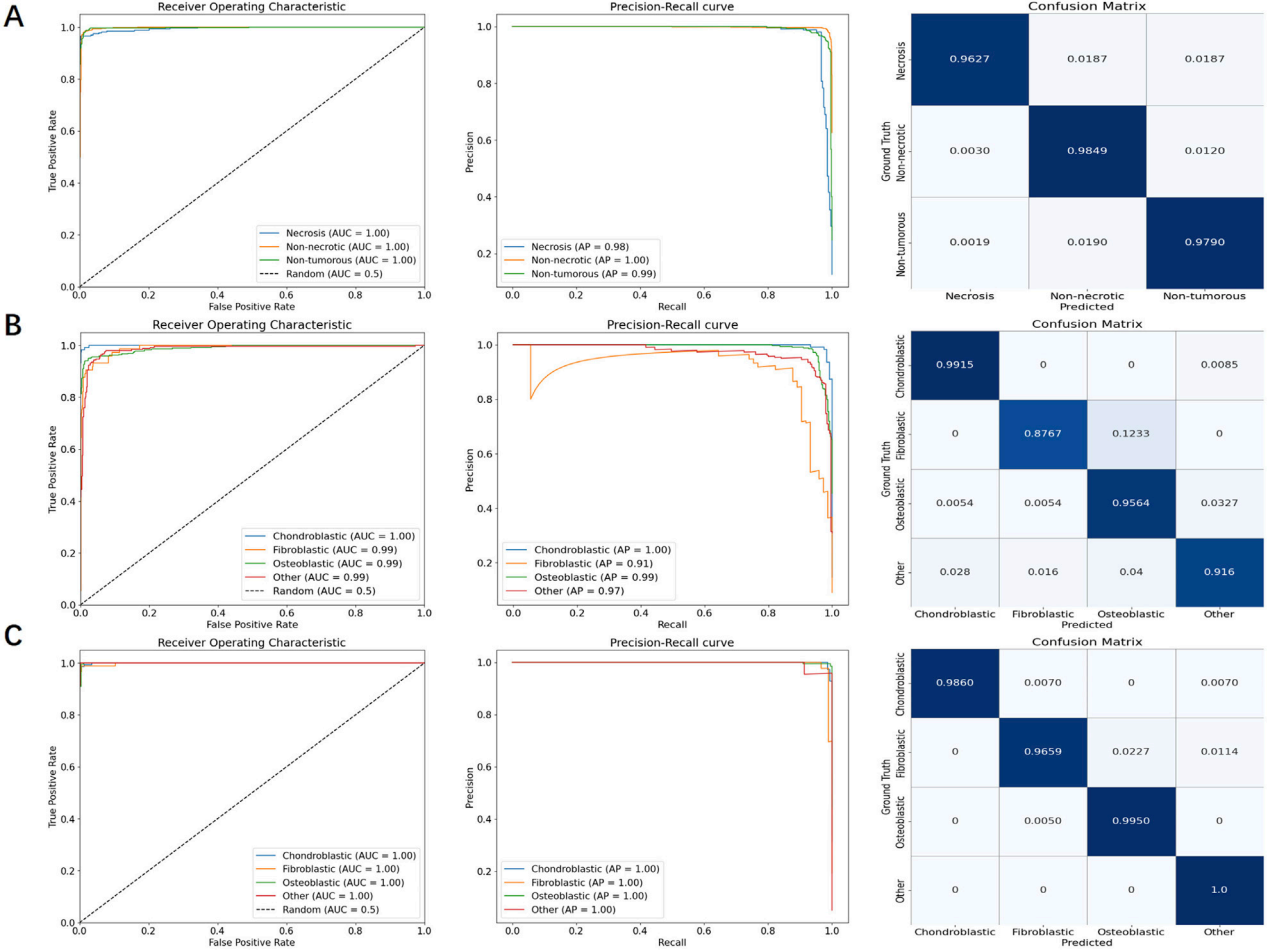
PR curves, ROC curves, and confusion matrices for necrosis classification, overall osteosarcoma subtypes, and localized osteosarcoma subtypes, respectively. **(A)** necrosis classification; **(B)** overall osteosarcoma subtypes; **(C)** localized osteosarcoma subtypes.

We used three models to classify necrosis, overall osteosarcoma subtypes, and localized osteosarcoma subtypes. All these models were based on the ViM architecture, with input images scaled to a fixed size of 512 pixels. The PR curve, ROC curve, and confusion matrix for each model are shown in Figure 6. As shown in Table 5, the AUC, sensitivity, specificity, PPV, and NPV for necrosis classification, overall osteosarcoma subtypes, and localized osteosarcoma subtypes all exceeded 90%.

**TABLE 5:** FP, FN, AUC, sensitivity, specificity, PPV, and NPV values for necrosis classification, overall osteosarcoma subtypes, and localized osteosarcoma subtypes.

Algorithm	Label	Total	TP	FP	FN	TN	Accur-acy	Recall	Precis-ion	F1	AP	AUC	Specif-icity	Sensiti-vity	PPV	NPV	TPR	FPR
Necrosis	Necrosis	268	258	5	10	1848	98.1%	96.3%	98.1%	97.2%	98.4%	99.5%	99.7%	96.3%	98.1%	99.5%	96.3%	0.3%
	Non-necrotic	1328	1308	15	20	778	98.1%	98.5%	98.9%	98.7%	99.8%	99.7%	98.1%	98.5%	98.9%	97.5%	98.5%	1.9%
	Non-tumorous	525	514	21	11	1575	98.1%	97.9%	96.1%	97.0%	99.5%	99.8%	98.7%	97.9%	96.1%	99.3%	97.9%	1.3%
overall subtypes (OS)	Chondroblastic	118	117	9	1	681	94.2%	99.2%	92.9%	95.9%	99.8%	100.0%	98.7%	99.2%	92.9%	99.9%	99.2%	1.3%
	Fibroblastic	73	64	6	9	729	94.2%	87.7%	91.4%	89.5%	90.9%	98.9%	99.2%	87.7%	91.4%	98.8%	87.7%	0.8%
	Osteoblastic	367	351	19	16	422	94.2%	95.6%	94.9%	95.3%	99.0%	99.0%	95.7%	95.6%	94.9%	96.3%	95.6%	4.3%
	Other	250	229	13	21	545	94.2%	91.6%	94.6%	93.1%	97.2%	98.5%	97.7%	91.6%	94.6%	96.3%	91.6%	2.3%
localized subtypes (OS)	Chondroblastic	143	141	0	2	310	98.7%	98.6%	100.0%	99.3%	99.9%	100.0%	100.0%	98.6%	100.0%	99.4%	98.6%	0.0%
	Fibroblastic	88	85	2	3	363	98.7%	96.6%	97.7%	97.1%	99.6%	99.9%	99.5%	96.6%	97.7%	99.2%	96.6%	0.5%
	Osteoblastic	199	198	2	1	252	98.7%	99.5%	99.0%	99.2%	99.9%	100.0%	99.2%	99.5%	99.0%	99.6%	99.5%	0.8%
	Other	23	23	2	0	428	98.7%	100.0%	92.0%	95.8%	99.6%	100.0%	99.5%	100.0%	92.0%	100.0%	100.0%	0.5%

In particular, the tumor region filtering model, introduced earlier, was used to classify necrosis. This model not only filters out small targets (classified as “other”) but also identifies the presence of necrosis within tumors. The ROC curve showed an AUC of approximately 99.5%, while the PR curve showed an AP value of around 98.4%. Additionally, the confusion matrix revealed very few misclassifications between different categories.

For the specific classification of osteosarcoma subtypes, due to sample size limitations, we only examined three types, namely, chondroblastic, fibroblastic, and osteoblastic subtypes. Other subtypes, as well as non-tumor samples, were categorized as “other”. We evaluated both overall subtype classification (based on the entire segmented tumor region) and localized osteosarcoma subtype classification (based on significant regions within the tumor). A comparison of the results shown in Table 5 revealed that the detection of localized osteosarcoma subtypes outperformed that of overall osteosarcoma subtypes. The results in Figure 6 reveal that fibroblastic osteosarcoma was more prone to be misclassified as osteoblastic in overall subtype classification, but this error was significantly reduced in localized osteosarcoma subtype classification. This finding indicates that incorporating local detail significantly improves the accuracy of osteosarcoma subtype classification.

As demonstrated in Table 5, subtype classification achieved generally satisfactory performance when evaluated using false positive (FP) and false negative (FN) rates. Notably, the fibroblastic subtype showed a disproportionately high false negative rate (9 cases), representing approximately 12% of its total samples in the classification analysis. The confusion matrix in Figure 6 reveals that the majority of these misclassified fibroblastic cases were incorrectly categorized as osteoblastic. This observation underscores a particular diagnostic challenge in distinguishing between fibroblastic and osteoblastic subtypes, which may stem from their shared morphological characteristics. These results indicate that future studies should prioritize 1. enhanced feature representation for fibroblastic subtypes and 2. expanded training datasets to mitigate classification errors.

### 3.3 Patient-level analysis

To determine the final osteosarcoma subtype at the patient level, we used two aggregation strategies: one based on the total area of each subtype and the other on the block count of each subtype. For the overall osteosarcoma subtype analysis, the total area of each subtype was summed up across all slides for each patient, and the subtype with the largest area proportion was assigned as the overall subtype of the patient. Conversely, the localized osteosarcoma subtype analysis summed up the block counts of each subtype across all slides, classifying the subtype with the highest block proportion as the localized osteosarcoma subtype of the patient.

A comparative evaluation revealed notable differences in the classification performance. The overall osteosarcoma subtype analysis achieved a classification accuracy of 91.7%, but higher misclassification rates were observed, particularly between chondroblastic and fibroblastic subtypes. In contrast, the analysis of localized osteosarcoma subtypes demonstrated superior performance, with a classification accuracy of 96.7%. The finer granularity of block-level aggregation provided a more precise representation of tumor heterogeneity, significantly reducing subtype misclassification.

**FIGURE 7:**
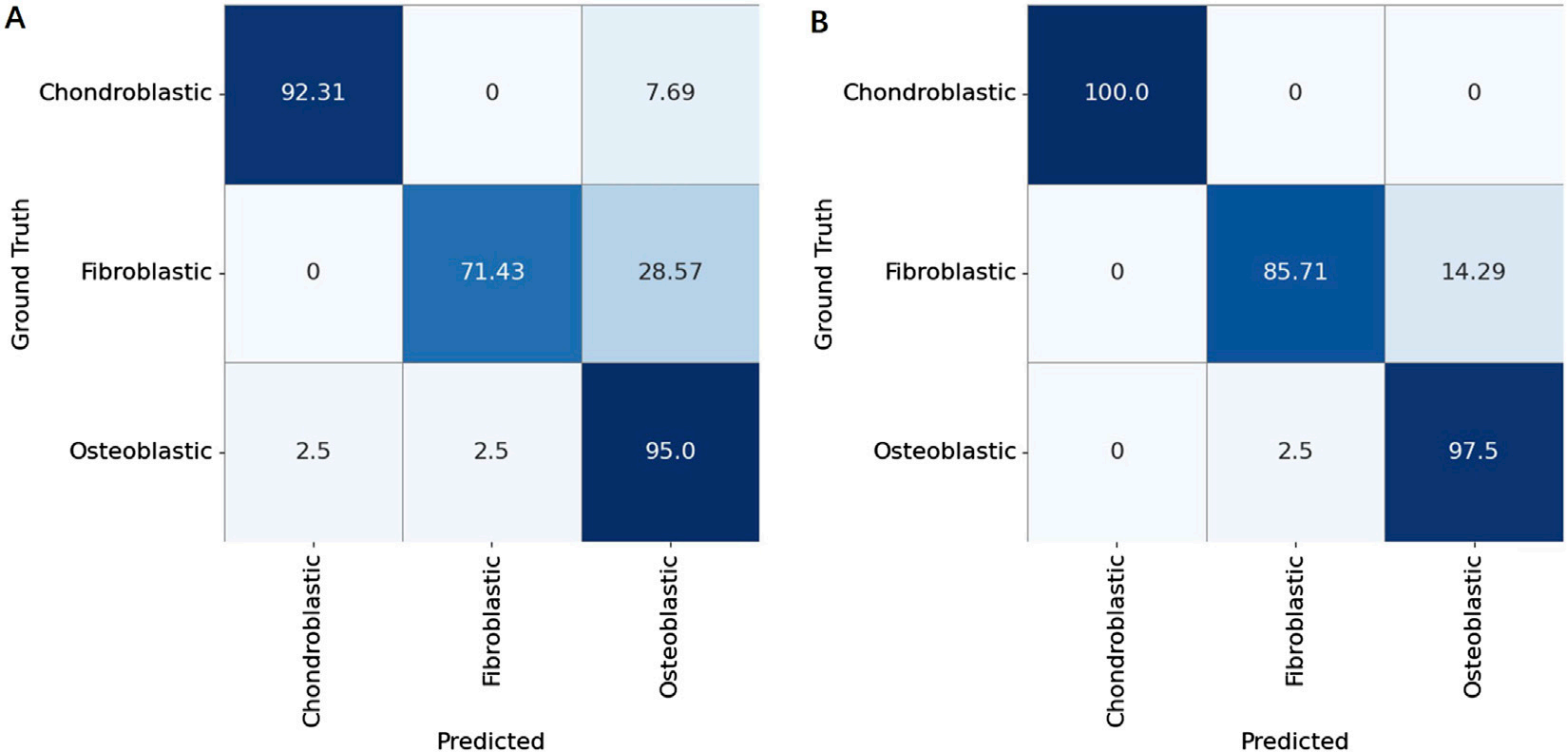
Confusion matrices for patient-level classification of osteosarcoma subtypes (Chondroblastic, Fibroblastic, and Osteoblastic) using two aggregation strategies. **(A)** Results obtained by aggregating tile-level predictions based on the overall area proportion of each subtype within a patient’s whole slide image. **(B)** Results obtained by aggregating tile-level predictions based on the local block count (majority voting of subdivided regions).

To validate these findings, we constructed confusion matrices for both methods (Figure 7), which revealed the classification performance of these methods. Patient-level aggregation, while integrating data from multiple slides for a holistic diagnosis, had a slightly lower accuracy than slide-level analysis. This discrepancy is due to the presence in certain cases of multiple coexisting subtypes within local regions, complicating subtype determination. Despite this, patient-level methods showed robust performance, emphasizing the importance of integrating multi-slide information for clinical workflows and offering potential for further refinement in osteosarcoma diagnostics.

## 4 Discussion

Osteosarcoma, a highly malignant and heterogeneous tumor primarily affecting children and adolescents, presents significant challenges in diagnosis and classification. Although current treatment modalities, such as neoadjuvant chemotherapy (NAC) and surgery, are the gold standard, evaluating therapeutic outcomes remains labor-intensive and complex. This study addresses these challenges by developing a comprehensive multimodal cascaded DL framework. By integrating advanced models, including the ViM network and ViM-UNet architecture, the framework systematically analyzes viable, necrotic, and non-tumor regions, alongside classifying tumor subtypes and identifying metastatic features. These advancements represent substantial improvements in diagnostic accuracy and efficiency.

### 4.1 Comparative analysis with previous work

Unlike previous studies that predominantly focused on isolated tasks, such as segmentation or classification at the patch level, this study uniquely combines case-level, whole-slide, and localized analyses within a unified pipeline. Earlier studies, such as those by Mishra et al. and Aziz et al. (29, 30), achieved commendable accuracy, but operated within constrained scopes—focusing on either segmentation or classification without addressing the whole complexity of osteosarcoma pathology. On the other hand, our cascading workflow integrates these tasks hierarchically, enabling a comprehensive and clinically actionable evaluation. This structured methodology significantly outperforms traditional patch-based models, setting a new standard for AI application in pathology (Table 6).

**TABLE 6:** Summary of similar sarcoma research studies.

Author	Year	Methodology	Accuracy (%)	Key features
B. S. Vandana et al. (36)	2020	Enhanced GraphCut-based clustering	90	Automated segmentation, multiclass classification using Random Forest
B. S. Vandana et al. (37)	2018	Object and color-based segmentation methods with SVM classification	93.7	Feature extraction, automated segmentation of tissue cells
Z. Li et al. (38)	2017	Machine learning classifiers (Random Forest, SVM, Logistic Regression)	95	Metabolomic data analysis with AUC 0.99
P. Bansal et al. (39)	2023	Multi-feature non-seed-based region growing segmentation	N/A	Improved ROI extraction using Marine Predators algorithm
K. V. Deepak et al. (40)	2023	Ensemble machine learning (color and texture feature extraction)	98.5	Accurate classification of bone tumors
B. Karthicsonia et al. (41)	2024	Multilayer grid XGBoost architecture combining ML and DL	N/A	High accuracy differentiation of normal and necrotic tissues
D. M. Anisuzzaman et al. (42)	2021	Deep learning with CNNs and transfer learning	96	Pre-trained CNNs (e.g., VGG19, Inception V3) for histological analysis
H. B. Arunachalam et al. (35)	2019	Machine learning and deep learning for tumor region assessment	N/A	Viable vs. necrotic tumor discrimination on WSIs
S. J. Badashah et al. (43)	2021	Fractional-Harris Hawks Optimization-based GAN	95.65	Improved accuracy, sensitivity, specificity
R. Mishra et al. (30)	2018	Convolutional Neural Network (CNN)	92	Efficient tumor classification using CNN architectures
R. A. Nabid et al. (44)	2020	Sequential Recurrent CNN with GRU	N/A	Handles heterogeneity and noisy data
M. D’Acunto et al. (45)	2019	Deep learning on microscopy images	N/A	Cell detection and classification
S. Alsubai et al. (46)	2024	Group Teaching Optimization with Capsule Network	N/A	Feature extraction with CapsNet and SA-BiLSTM
M. T. Aziz et al. (29)	2023	Hybrid CNN and MLP framework	95.2	Binary and multiclass classification with decision tree-based RFE
Y. Fu et al. (47)	2020	Deep model with Siamese Network (DS-Net)	95.1	Viable and necrotic tumor classification
S. Prabakaran et al. (48)	2023	Hyperparameter-tuned Elman Neural Network (HTDENN)	95.31	High precision, sensitivity, and accuracy
M. A. A. Walid et al. (49)	2023	Deep ensemble learning with CNN models	N/A	High Kappa score with voting classifier
J. Wu et al. (50)	2023	Semantic segmentation with ENMViT	N/A	Precise segmentation in resource-limited settings

### 4.2 Limitations and challenges

Despite its advancements, this study has several limitations:

1. Limited case-level samples: Osteosarcoma is a rare and heterogeneous malignancy, and both institutional and publicly available datasets are extremely limited. Our study combined a retrospective cohort from Chongqing General Hospital (2012–2022) and the public UT Southwestern dataset (1995–2015) to maximize sample diversity. We recognize that such pooling may introduce batch effects due to differences in staining protocols, scanning devices, and patient demographics. This remains one of the limitations of our work.
2. Regression model feasibility: Although the framework provides valuable quantitative outputs, building intelligent regression models for case-level analysis demands extensive sample sizes. The current dataset is insufficient to support the development of robust regression models.
3. Limitations of the pathological subtype classification: This study included some post-chemotherapy samples. Chemotherapy can lead to changes in the morphology of tumor cells, including alterations in size, shape, nuclear condensation, and reduced cytoplasm. At the same time, chemotherapy drugs can also affect the tumor stroma. After chemotherapy, the tumor stroma often exhibits fibrosis, foam cell reaction, and lymphocyte infiltration. In some cases, large areas of necrosis can be observed in tumors following chemotherapy. We study primarily focused on the three main subtypes of osteosarcoma. The number of pathological samples is limited, and some patients received preoperative chemotherapy, which influenced the morphology of tumor cells and introduced bias to the results. Additionally, a detailed analysis of less common subtypes was not performed, leading to an insufficient examination of the heterogeneity of osteosarcoma. However, fortunately, a significant area of necrosis was observed in the tumors of some patients who underwent chemotherapy. Our model identified the necrotic areas, which will be helpful for future assessments of chemotherapy effectiveness in these patients.
4. Subtype-specific classification strategy: To address osteosarcoma heterogeneity, this study employed both area-weighted and tile-level classification approaches. The tile-level method—based on majority voting of fixed-size patches proved more effective in detecting small, spatially confined subtypes. Compared to area-weighted statistics that may overlook focal high-grade components, the tile-based strategy enhances sensitivity to local variations and better reflects mixed histological patterns. This was especially evident in chondroblastic and fibroblastic subtypes, where improved classification performance was observed.
5. Limitations of metastatic samples: This study included some lung metastasis samples. Osteosarcoma primarily metastasizes through the bloodstream, with the lungs being the most common site of metastasis. During the metastatic process, it is possible that only a portion of the tumor cells spread. Due to the heterogeneity within the tumor, the metastatic tumor may exhibit different histological features compared to the primary tumor. Additionally, the microenvironment surrounding the metastatic tumor differs from that of the primary tumor. A combination of various factors contributes to the distinct pathological characteristics of the metastatic tumor. However, this study aimed to demonstrate the superiority of the cascade model within a workflow; whether lung metastasis has occurred is one of the branch outputs. Additionally, this study compared the effect of osteoid matrix between bone samples and lung metastasis samples and found that the model can still accurately identify osteoid matrix in lung metastasis samples. Therefore, lung metastasis samples were ultimately retained.
6. Clinical Integration: The absence of extensive clinical trials and real-world validation restricts the immediate applicability of the framework in medical practice. Prospective studies in clinical environments are critical to bridge this gap.

### 4.3 Future directions

To overcome the above limitations and expand the scope of the current study, the following areas will be prioritized in future research:

1. Dataset expansion: Acquiring larger and more diverse datasets will enhance model robustness and enable more generalized conclusions. This will also facilitate the development of regression models for predicting clinical outcomes based on case-level quantitative indicators.
2. Comprehensive subtype analysis: Broadening the analysis to include additional rare osteosarcoma subtypes will provide a more holistic understanding of the disease and its variations.
3. Prognostic modeling: Enhancing the accuracy of the model in identifying necrotic areas, and integrating this framework with prognostic tools like DeepSurv to predict patient outcomes and guide treatment strategies (31–35).
4. Clinical validation and workflow integration: Conducting extensive clinical trials to validate the performance of the framework in real-world settings and integrating it into clinical workflows to streamline diagnostic processes.
5. Self-supervised learning approaches: Reducing reliance on manual annotations by using unsupervised or self-supervised learning techniques, thereby enhancing scalability and adaptability.

### 4.4 Conclusion

This study represents a significant advancement in leveraging AI for the pathological analysis of osteosarcoma. By addressing the heterogeneity and complexity issues of osteosarcoma through a multimodal cascaded framework, this study enhances diagnostic precision while setting the stage for integrating AI-driven solutions into clinical workflows. Future work focusing on dataset expansion, subtype diversity, and clinical validation will ensure these advancements translate effectively into improving patient care and outcomes.

## Data Availability

The raw data supporting the conclusions of this article will be made available by the authors, without undue reservation.
