# Supplementary material for: Research on the application of a multi-model cascaded deep learning framework in the pathological diagnosis of osteosarcoma
Source: Oncol Rev. 2025 Nov 12;19:1592408. doi: 10.3389/or.2025.1592408 (PMC12646879; doi:10.3389/or.2025.1592408)
Supplement: Supplementary file 1 [file Supplementaryfile1.docx]

**Supplementary**

**Text S1. Model Training Protocols and Pathology-Specific Augmentation Strategies**

**1. Standardized Training Configuration**

All segmentation and classification models were trained under a unified configuration framework to ensure reproducibility and comparability across experiments.

**(1) Optimizer:** All models employed the Adam optimizer, with a learning rate of 0.001, weight decay of 0.0001, betas of (0.9, 0.999), and epsilon set to 1e-8.

**(2) Learning Rate Scheduling:** A linear warm-up strategy was applied during the first 100 iterations (warmup ratio = 0.1), followed by a cosine annealing decay (minimum learning rate ratio = 0.05).

**(3) Training Epochs:** Segmentation models were trained for 100 epochs, while classification models were trained for 50 epochs.

**(4) Runner and Logging:** The EpochBasedRunner framework was adopted with checkpointing at each epoch (maximum of 1 checkpoint retained), logging every 10 iterations, and real-time monitoring via TensorBoard.

**(5) Distributed Training:** All models were trained using PyTorch’s distributed learning infrastructure with NCCL backend for inter-GPU communication.

**2. Transfer Learning and Backbone Initialization**

All models were constructed based on the Vision Mamba (ViM) architecture. Two backbone variants were used: ViM-Base for classification tasks and ViM-UNet for segmentation tasks.

Initial weights for both backbones were derived from public ImageNet-1K pretrained checkpoints. To address staining variation and adapt to histological heterogeneity, selected models were further fine-tuned using approximately 20,000 internal tiles extracted from osteosarcoma datasets. This fine-tuning aimed to better align the feature distributions with the domain-specific data encountered during downstream tasks.

**3. Augmentation and Sampling Pipeline for Segmentation Tasks (ViM-UNet)**

A pathology-aware augmentation framework was implemented to accommodate the complexity of high-resolution whole slide images (WSIs). The pipeline comprises the following steps:

**(1) Region-of-Interest Sampling:** For each training epoch, a randomly selected expert-annotated region (e.g., tumor, necrosis, osteoid matrix) from the WSI was used as the basis for tile extraction.

**(2) Scale Normalization:** Each WSI was rescaled to a consistent physical resolution based on task-specific micron-per-pixel (mpp) values (e.g., 5.0, 2.0, or 0.23 µm/pixel), minimizing the influence of microscope imaging variability.

**(3) WSI-Level Rotation:** Rotations were performed directly at the WSI level prior to cropping. This approach avoids black edge artifacts and ensures the spatial continuity and integrity of tissue pixels within each tile.

**(4) Perturbed Tile Extraction:** Tiles of 1024×1024 pixels were sampled from the rotated region, incorporating random scale perturbations (0.8–1.2x) and translational jitter (±128 px). Approximately 100 tiles were generated per WSI in each epoch.

**(5) Tissue Masking:** Non-informative or background regions (e.g., blank slide areas or artifacts) were masked to zero to suppress irrelevant signals during training.

This augmentation method differs from traditional post-cropping rotation, enabling higher spatial fidelity and maximizing usable image context. It is particularly effective for tasks involving small or heterogeneous histological structures.

**4. Augmentation and Sampling Pipeline for Classification Tasks (ViM-Base)**

For classification models involving necrosis detection or subtype classification, a segmentation-guided and geometry-aware tile generation strategy was utilized. The process was defined as follows:

**(1) Contour Extraction:** Polygonal contours delineating pathological structures of interest (e.g., tumor, necrotic foci) were extracted from prior segmentation predictions.

**(2) Global Rotation:** As with segmentation, rotation was conducted on the full WSI level before tile extraction, avoiding spatial discontinuity at tile borders.

**(3) Contextual Expansion:** The bounding box of each region was isotropically expanded by a predefined factor (e.g., 1.2×) to retain sufficient morphological context for classification.

**(4) Tile Sampling with Perturbation:** Tiles of fixed input size (e.g., 448×448 or 512×512 pixels) were cropped from the expanded bounding box, incorporating slight random scaling (0.9–1.1x) and translation (±32 px).

**(5) Target Masking:** Pixels outside the annotated contour were zero-masked to prevent leakage of irrelevant context and reinforce the model's attention to the designated lesion.

This composite strategy integrates anatomical priors, contextual learning, and robust augmentation. It is particularly beneficial for complex diagnostic settings such as osteosarcoma subtype classification, where intra-class variability is high and histological boundaries are subtle.

**Text S2. Analysis of Dice Sensitivity to Small Target Volume and Multi-Metric Evaluation Strategy**

In this study, we observed that certain pathological subtypes (e.g., chondroblastic, fibroblastic, and other rare osteosarcoma subtypes) exhibited lower Dice coefficients in segmentation tasks, despite relatively high values in other metrics such as Accuracy and Specificity. To further investigate this phenomenon, we conducted a theoretical analysis of Dice sensitivity to target size, supported by multi-metric validation.

Let $G\subset R^{2}$ denote the ground truth region and $P\subset R^{2}$ the predicted region. The Dice coefficient is defined as:

$$\mathrm{Dice}\left( P, G \right)=\frac{2\left| P\cap G \right|}{\left| P \right|+\left| G \right|}$$

Assuming prediction errors arise from boundary shifts and lead to symmetric differences $E=\left( P\backslash G \right)\cup\left( G\backslash P \right)$, the error area is denoted $\left| E \right|=\varepsilon$. Let us assume the predicted region has the same total area as the ground truth ($\left| P \right|=\left| G \right|$), then the intersection area becomes $\left| P\cap G \right|=\left| G \right|-\varepsilon/2$. Substituting into the Dice formula yields:

$$\mathrm{Dice}\left( P, G \right)=\frac{2\left( \left| G \right|-\varepsilon/2 \right)}{2\left| G \right|}=1-\frac{\varepsilon}{2\left| G \right|}$$

This indicates that when the prediction error $\varepsilon$ is constant, the Dice coefficient decreases inversely with the size of the ground truth region $\left| G \right|$. Consequently, smaller targets are more susceptible to apparent performance degradation under Dice evaluation, even when the absolute error is minimal.
 To address this bias in small-target evaluation, we performed a comprehensive analysis using multiple evaluation metrics. For example, as shown in Table 2, although the Dice scores for chondroblastic and other rare subtypes were relatively lower (0.7184 and 0.7166, respectively), the models still achieved high Specificity (99.87%, 99.98%) and Accuracy (99.26%, 99.88%), along with balanced Precision values (88.39%, 89.73%). These findings indicate that the model reliably distinguishes these subtypes despite the Dice limitation.

Table S1. Detailed Configuration of Training Parameters for Each Model.

| **Model Name** | **Task Type** | **Image Input Strategy** | **Image Scaling Parameter (mpp or Size)** | **Optimizer** | **Learning Rate Strategy** | **Total Epochs** |
| --- | --- | --- | --- | --- | --- | --- |
| Tumor Region Segmentation | Segmentation | Tile extraction + large-tile cropping | mpp = 5.0 | Adam, lr=1e-3, wd=1e-4 | Linear warmup + Cosine decay | 200 |
| Osteoid Matrix Segmentation | Segmentation | Tile extraction + large-tile cropping | mpp = 2.0 | Same as above | Same as above | 200 |
| Necrotic Area Segmentation | Segmentation | Tile extraction + large-tile cropping | mpp = 2.0 | Same as above | Same as above | 200 |
| Tumor Cell Segmentation | Segmentation | Tile extraction + small-tile sampling | mpp = 0.23 | Same as above | Same as above | 200 |
| Lung Metastasis Segmentation | Segmentation | Tile extraction + large-tile cropping | mpp = 5.0 | Same as above | Same as above | 200 |
| Necrosis Classification | Classification | Whole-image resizing | 512×512 px | Adam, lr=1e-3, wd=1e-4 | Linear warmup + Cosine decay | 100 |
| Overall Subtype Classification | Classification | Whole-image resizing | 512×512 px | Same as above | Same as above | 100 |
| Localized Subtype Classification | Classification | Large-tile aggregation + statistical analysis | 384×384 px | Same as above | Same as above | 100 |

Table S2. Detailed Configuration of Each Module in the Cascaded Workflow.

| **Module Name** | **Magnification (mpp)** | **Input Size (pixels)** | **Model Type** | **Output Result** | **Gating Logic** |
| --- | --- | --- | --- | --- | --- |
| Effective Area Segmentation | 5.0 µm/pixel | 1024×1024 | ViM-UNet | Tissue area mask | score > 0.6, area > 1000 pix |
| Tumor Region Segmentation | 5.0 µm/pixel | 1024×1024 | ViM-UNet | Tumor region mask | score > 0.6, area > 1000 pix |
| Lung Metastasis Tumor Region Segmentation | 5.0 µm/pixel | 1024×1024 | ViM-UNet | Metastatic tumor region mask | score > 0.6, area > 1000 pix |
| Tumor Region Filtering/Necrosis Classification | - | 512×512 | ViM-Base Classifier | Valid tumor ROI tile | score > 0.8 |
| Tumor Cell-Significant Region Detection | 2.0 µm/pixel | 1024×1024 | ViM-UNet | High-density tumor cell regions | score > 0.6, area > 1000 pix |
| Osteoid Matrix Segmentation | 2.0 µm/pixel | 1024×1024 | ViM-UNet | Osteoid matrix mask | score > 0.6, area > 1000 pix |
| Necrotic Region Segmentation | 2.0 µm/pixel | 1024×1024 | ViM-UNet | Necrotic region mask | score > 0.6, area > 1000 pix |
| Overall Subtype Classification | - | 512×512 | ViM-Base Classifier | Dominant subtype (area-weighted) | score > 0.8 |
| Localized Subtype Classification | - | 384×384 | ViM-Base Classifier | Local dominant subtype (tile count-based) | score > 0.8 |
| Osteoid Matrix Removal | - | - | Logic Module | Tumor region after osteoid matrix removal | - |
| Tumor Cell Segmentation | 0.23 µm/pixel | 1024×1024 | ViM-UNet | Pixel-level tumor cell mask | score > 0.4, area > 100 pix |
| Lung Metastasis Osteoid Matrix Segmentation | 2.0 µm/pixel | 1024×1024 | ViM-UNet | Osteoid matrix in metastatic foci | score > 0.6, area > 1000 pix |
